# A validation of the Malaria Atlas Project maps and development of a new map of malaria transmission in Sokoto, Nigeria: a cross-sectional study using geographic information systems

**DOI:** 10.1186/s12936-020-03214-8

**Published:** 2020-04-08

**Authors:** Usman Nasir Nakakana, Ismaila Ahmed Mohammed, B. O. Onankpa, Ridwan M. Jega, Nma Muhammad Jiya

**Affiliations:** 1 Department of Paediatrics, Usmanu Danfodiyo University Teaching Hospital, Sokoto, Nigeria; 2 Medical Research Council, The Gambia at London School of Tropical Medicine and Hygiene, Fajara, The Gambia; 3 Department of Community Medicine, Usmanu Danfodiyo University Teaching Hospital, Sokoto, Nigeria

**Keywords:** Malaria, Endemicity, Malaria Atlas Project, Mapping, Nigeria, Geographic information systems

## Abstract

**Background:**

Malaria remains a major cause of morbidity and mortality among children in Africa. There is inadequate information regarding malaria transmission-intensity in some of the worst-affected parts of sub-Saharan Africa (SSA). The Malaria Atlas Project (MAP) was developed in 2006, to project estimates of malaria transmission intensity where this data is not available, based on the vector behaviour for malaria. Data from malariometric studies globally were obtained and modelled to provide prevalence estimates. The sensitivity of these maps, however, reduces with unavailability of data. This necessitates a validation of these maps locally, and investigation into alternative methods of predicting prevalence to guide malaria control interventions and improve their efficiency and effectiveness. This study was conducted to compare the true estimates in Sokoto, Nigeria, with the MAP projections for north-western Nigeria, and it proposes an alternative way of mapping malaria intensity in Nigeria and beyond.

**Methods:**

A malariometric survey was conducted including children aged 2–10 years in communities in Wamakko Local Government Area (LGA) of Sokoto State, Nigeria. Children had blood examinations for the presence of malaria parasitaemia and a physical examination for the signs of clinical malaria. All the sites from which children were included in the study were geo-located using a hand-held Global Positioning System (GPS) device and compared this to MAP maps of the same area. A mapping software was also used to generate a malaria prevalence map of the study area, considering the average flight distances of the vector.

**Results:**

The prevalence among children 2 to 10 years was found to be 34.8% which was within the 30–40% projected prevalence for the study area by MAPs. However, it was much lower than the projection during the dry season (20.2%) and higher than the projected estimate during the rainy season (49.3%). There was monoparasitaemia of *Plasmodium falciparum* throughout the study area, although the study was not specifically designed to identify other species. The prevalence of parasitaemia and splenomegaly were similar when overall and when considered by age of the participants. The study also generated a map of malaria transmission, which mapped out areas where the prevalence was confirmed or likely to be to be within the range of 30–40%, based on the sites which constituted the study area for this study.

**Conclusion:**

The study concludes that the prevalence of malaria and its transmission intensity in Sokoto are similar to Malaria Atlas Project predictions for the area and that, for malaria control planning purposes, the projections may be utilized, with more efforts at validation of the MAPs in other locations and terrains. Also, the vector behaviour may be used to map transmission of malaria and other vector-transmitted diseases, where this information is lacking.

## Background

Malaria is a preventable disease with a widespread distribution and high public health impact. In 2017, about 219 million cases of malaria occurred worldwide [1]. 97 countries and territories have ongoing malaria transmission, with an estimated 3.2 billion people said to be at risk of the disease. Of these, 1.2 billion are at high risk, implying that more than one malaria case occurs per 1000 population [1, 2]. Africa is disproportionately affected by malaria deaths, accounting for about 93% of all malaria deaths, with 19% of the global deaths in Nigeria. Estimates showed that about 266,000 African children died before their fifth birthday in 2017 from malaria, and although there is inadequate data from disease notification from Nigeria, an estimated 177.5 million cases of malaria occurred in 2015 [3, 4]. The disease is transmitted by the female *Anopheles* mosquito which sustains the cycle of transmission. Due to the behaviour of the vector, the disease transmission is ‘local and focal’ and dependent on the flight range of the mosquito. It is also affected by geographical factors such as altitude, vegetation, topography, rainfall and seasonality [5].

The intensity of malaria transmission or endemicity has been described based on the prevalence of peripheral malaria parasitaemia in children 2 to 10 years of age (PfPR_2–10_). This is because the index has been found to closely correlate with the entomological inoculation rate (EIR) which refers to the number of infectious bites per person per day [6].

Since the dawn of the twenty-first century, there has been an explosion in the volume of available data on global parasite rates [7]. For this reason, as well as the challenges of repeated prevalence estimation, an initiative in 2006 was developed which was the Global Malaria Atlas Project system (MAPs). It sought to project the expected prevalence and endemicity of malaria in all locations around the world, by modelling the available prevalence data [8]. Due to the ability of PfPR_2–10_ to mirror the EIR, the models were created to predict the PfPR_2–10_ for a given set of environmental conditions. MAPs used data points generated from over 4800 malaria surveys for its estimation [9], combining this with GIS data. The basis for the estimation stems from the vector-transmitted nature of the disease which is very sensitive to climate [10]. The project utilized multiple sources of information including annual parasite incidence (API) data from countries, WHO regional offices, localized surveys as well as expert opinion to arrive at the conclusive data. The sensitivity of these MAPS, however, increases with abundance of prevalence data, considering that these data points are unevenly distributed. For Nigeria, PfPR_2–10_ data was obtained from six general locations in the country [8], but this is grossly insufficient for any meaningful mapping.

MAPs sought to improve on earlier prediction models which had not been validated and needed a standardization of the available prevalence rates to reflect the age group of 2 to 10 years [11]. The map was updated in 2010, including new malariometric data extending to 13,449 administrative units in 43 endemic countries and 22,212 *Plasmodium falciparum* parasite rate (PfPR) surveys were used to define spatial limits of malaria transmission. MAP thus gives a point estimate based on the available information, but fails to represent the seasonal nature of malaria infections. This hampers intervention planning including the timing of interventions such as seasonal malaria chemoprophylaxis, environmental control and when to distribute Insecticide Treated Nets (ITNs) because these interventions are cost-effective when deployed during the peak of malaria transmission, which is usually during the wet season. Additionally, considering that malaria transmission is focal and local, the big-picture data presented by MAPs is limited in usefulness for micro planning of malaria interventions and the deployment of local resources for malaria control. It may be useful, however when data is not available if the predictions are likely to be close to the reality.

Another more localized attempt to map malaria endemicity was the West African map also carried out using modelling techniques with data from 450 data points for studies conducted from 1970 onwards in African countries [12]. This map, based on the continental database of malaria survey results (MARA/ARMA) undertook a spatial analysis of malaria prevalence in relation to environmental factors involved in malaria transmission. It described 52.7% of the total West African population as being with a predicted prevalence of 30–70%, with 14.8% having an expected prevalence of 10–30% [12]. The class assignment, however, is not based on endemicity classification or the control-related classification and is thus of limited value. It was not built upon probably because of limitations in the availability of prevalence data such as the exclusion of urban populations from the model due to a paucity of data from those areas.

Considering that about 2.2 billion US Dollars was expended on malaria control activities in the World Health Organization (WHO) African region in 2017 alone, there is a need to maximize the efficiency of use of these resources by guiding interventions with data. While MAPs may provide some data, these predictions need to be tested in areas where there is particularly limited prevalence information. Recognizing that the areas of greatest need for malaria intervention are among the areas with the least information available. Additionally, there is a need to continually find new ways to more accurately predict the disease patterns for knowledge-guided intervention.

This study aimed to determine the endemicity of malaria in Sokoto state using the prevalence rate of *P. falciparum* among children 2–10 years of age in Wamakko Local Government Area, compare this to the MAP projection, and generate an alternative map for the study area.

## Methods

The specific objectives were: (i) to determine the prevalence of malaria parasitaemia among children 2 to 10 years in Wamakko LGA of Sokoto state; (ii) to compare the prevalence with MAPs prediction and map the intensity of malaria transmission predictions and identify factors responsible for any observed difference.

### Study setting

The study was conducted in Wamakko Local Government Area (LGA) of Sokoto State, located in North Western Nigeria; it has an area of 732.146 km^2^ and a population of 234,860 in 2017. It is located at coordinates 13° 2′ 16″ N 5° 5′ 37″ E [13]. The geography of the area is predominantly flat plains with Sudan Savannah type vegetation at an altitude of 292 m above sea level. Its climate is tropical, described as local steppe [14, 15].

### Study design

The study was a two-point, cross-sectional observational study conducted once in the rainy season in August 2016 and once in the dry season in December 2016. At least 422 subjects were needed based on the assumptions of a 70% response rate and that 80% of households include at least one child less than 5 years of age in accordance with previous malaria indicator surveys (MIS) in Nigeria [16]. For each of the seasons, at least 500 subjects were recruited for the study.

### Sampling technique

As is the norm for malaria indicator surveys [16], multistage cluster sampling in proportion to size was employed. Approximately 892 households were required in all to meet the target of at least 500 subjects per season. All children in the selected settlements who met the age criteria of 2 to 10 years, with or without symptoms of malaria were recruited for the study, provided they meet all the inclusion criteria, in the absence of exclusion criteria. The study included children aged 2 to 10 completed years and had a history of residence in the study area for at least 2 weeks prior to recruitment. All children who had taken anti-malarial medication and medication with anti-malarial activity, such as macrolides and clindamycin within the 2 weeks preceding the study were excluded.

### Procedures

Potential subjects were screened for eligibility and the caregivers of eligible subjects were required to sign informed consent forms. A Case report form (CRF) was used to collect information regarding the child and household. Each child had a physical examination, conducted by the lead investigator and recorded the findings accordingly. The results of malaria testing were also recorded on the CRF, once available. Palpable splenomegaly was assessed using Hackett’s method [17]. Participants with severe malaria received an initial intramuscular dose of artesunate before being promptly referred to the nearest tertiary hospital. The severity of malaria was determined using the WHO criteria for severe malaria [18].

Malaria sampling was with thick and thin films. Thick films were used to estimate parasite density, and thin films were for species identification. Rapid diagnostic tests (RDTs) (CareStart^®^ Malaria HRP2, Access Bio, Inc., model G0141), which can detect *P. falciparum* infections, were used for the purpose of treating sick participants quickly. The samples were analysed in a completely anonymized manner in pairs of thick and thin films. All thick films which were positive for malaria parasitaemia had their corresponding thin films examined for species of malaria parasite. For determination of the species distribution for clinical malaria, however, only the thin film results of individuals with clinical symptoms and signs of malaria infection were considered. At least 10 fields were examined before a slide was declared negative for malaria parasites [19]. Children with fever confirmed by RDT to have uncomplicated malaria were treated at home with artemisinin-based combination therapy (artemether–lumefantrine) [20]. All the selected sites for the study were geo-located using a hand-held GPS device, these points were plotted on a base map of the LGA and plotted as areas where the obtained prevalence exits. The area extending up to 5 km surrounding the study sites was plotted as areas with confirmed prevalence obtained from the study, with a penumbra another 5 km beyond there designated as areas with a likely prevalence of based on the average flight distances of the mosquito vector [21].

### Data analysis

The data was analysed using Stata^®^ version 15. The prevalence of malaria parasitaemia was calculated for the entire study population and relative proportions of the *Plasmodium* species causing parasitaemia. Subset analysis was for prevalence by age, gender and season. The prevalence obtained was compared with the prediction for Sokoto by the MAPs project. To get a balanced projection of the prevalence across the state, the parameters of temperature, rainfall, presence or otherwise of flooding, and the geography and terrain of the sites selected were compared, with other locations in the state, using the global malaria MAPS model. The climatological data used was sourced from climatedata.org [15].

## Results

### Subjects included

**Fig. 1:**
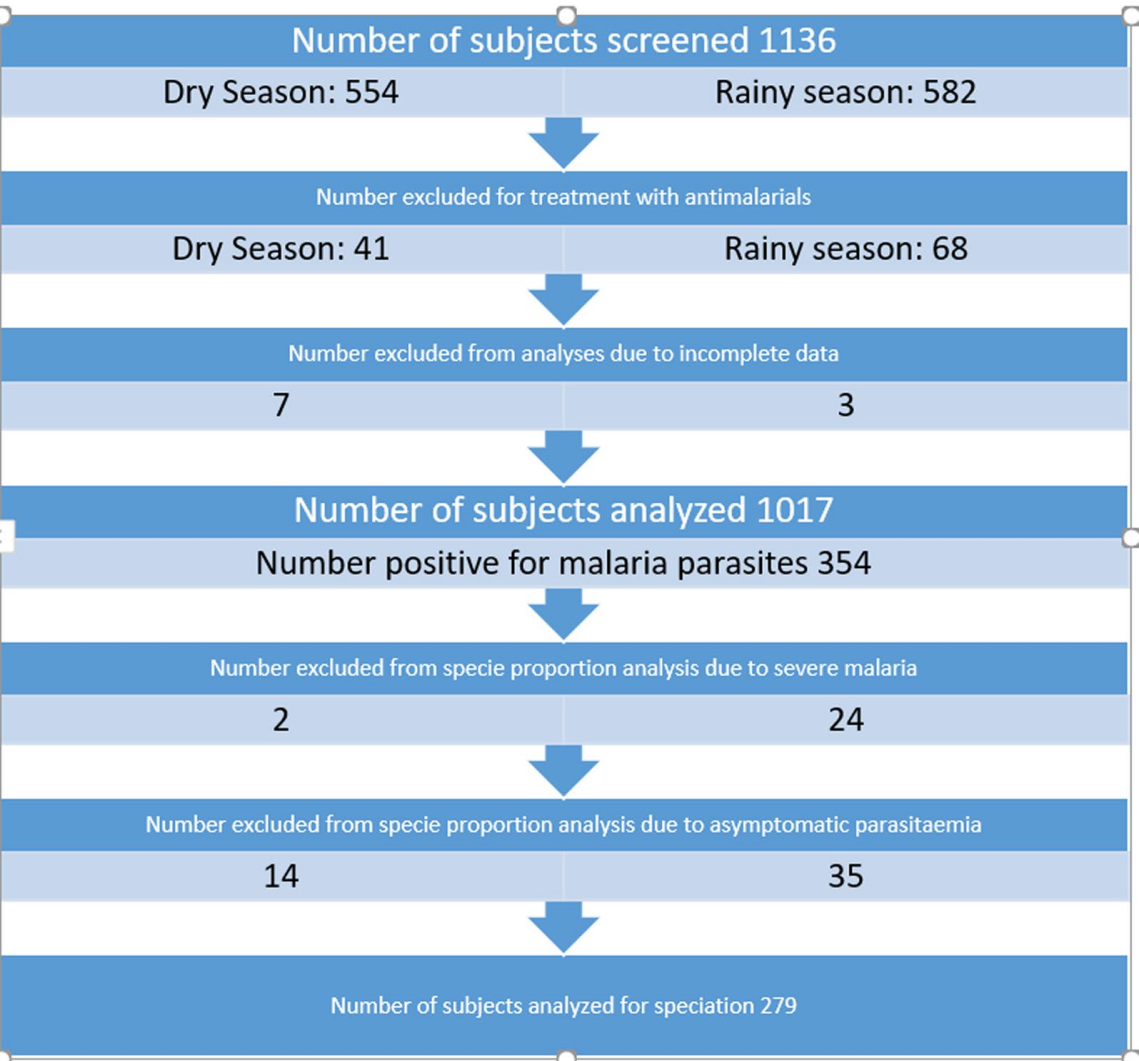
Diagram showing sequential subject recruitment

A total of 1136 subjects were screened for inclusion in the study after consenting (Fig. 1). 109 were excluded due to having been treated with anti-malarials in the 2 weeks prior to enrolment and 10 were excluded due to incomplete data. 1017 subjects were included in the analysis.

### Age and gender distribution of respondents

There were 525 males (51.6%) and 492 females (48.4%) with a male to female ratio of 1.1:1. The highest proportion of subjects were those aged 2 years (12.1%) compared with other ages and the least number were those aged 5 years (10.5%). The differences were however not statistically significant (p = 0.1) (Table 1).

**Table 1:** Age and gender distribution of the subjects included in the study

Age (completed years)	N	Gender	
		Male No (%)	Female No (%)
2	123	60 (48.8)	63 (51.2)
3	117	63 (53.8)	54 (46.2)
4	110	54 (49.1)	56 (50.9)
5	107	52 (48.6)	55 (51.4)
6	111	62 (55.9)	49 (44.1)
7	111	73 (65.8)	38 (34.2)
8	110	50 (45.5)	60 (54.5)
9	113	54 (47.8)	59 (52.2)
10	115	57 (49.6)	58 (50.4)
Total	1017	525 (51.6)	492 (48.4)

### Prevalence of malaria parasitaemia

The overall prevalence of malaria for the study was 34.8% with 95% confidence intervals of 31.9% and 37.8%, using microscopy and 33.8% with confidence intervals of 30.9% and 36.8% using RDT as shown in Table 2. There was an agreement between the two diagnostic methods as shown by the kappa statistic (p < 0.001).

**Table 2:** Prevalence of malaria parasitaemia among children 2–10 years using microscopy and RDT and their 95% confidence intervals

	Thick film	RDT
Positive	354	344
Negative	663	673
Prevalence	34.8%	33.8
Lower bound 95% CI	31.9	30.9
Upper bound 95% CI	37.8	36.8

Kappa agreement κ = 0.764, p < 0.001

### Parasite species distribution

The only species of *Plasmodium* parasite detected in the study across the study locations was *P. falciparum,* implying that the observed prevalence is the same as the PfPR2–10.

### Prediction across Sokoto state using MAPs model and difference in seasonal parasite rates

**Fig. 2:**
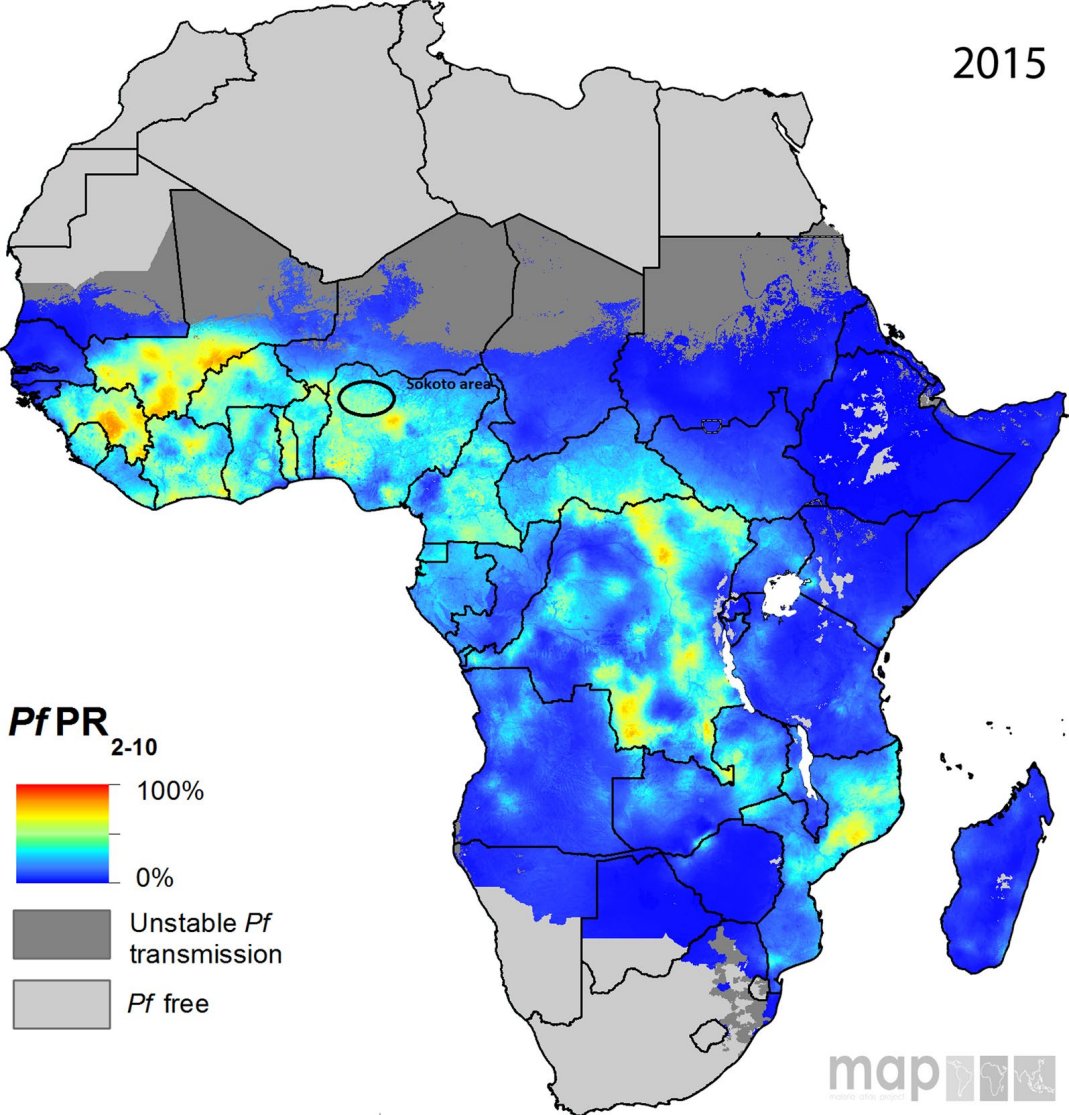
Maps data from 2015 with Sokoto area highlighted [23]

The obtained prevalence was similar to MAPs prediction, therefore the prevalence of malaria in Wamakko may be extrapolated to the whole of Sokoto state. Considering that Sokoto projected as homogenous in terms of prevalence and endemicity by MAPs as shown in Fig. 2.

### Mapping the prevalence of Malaria across Sokoto using PfPR2–10 data

**Fig. 3:**
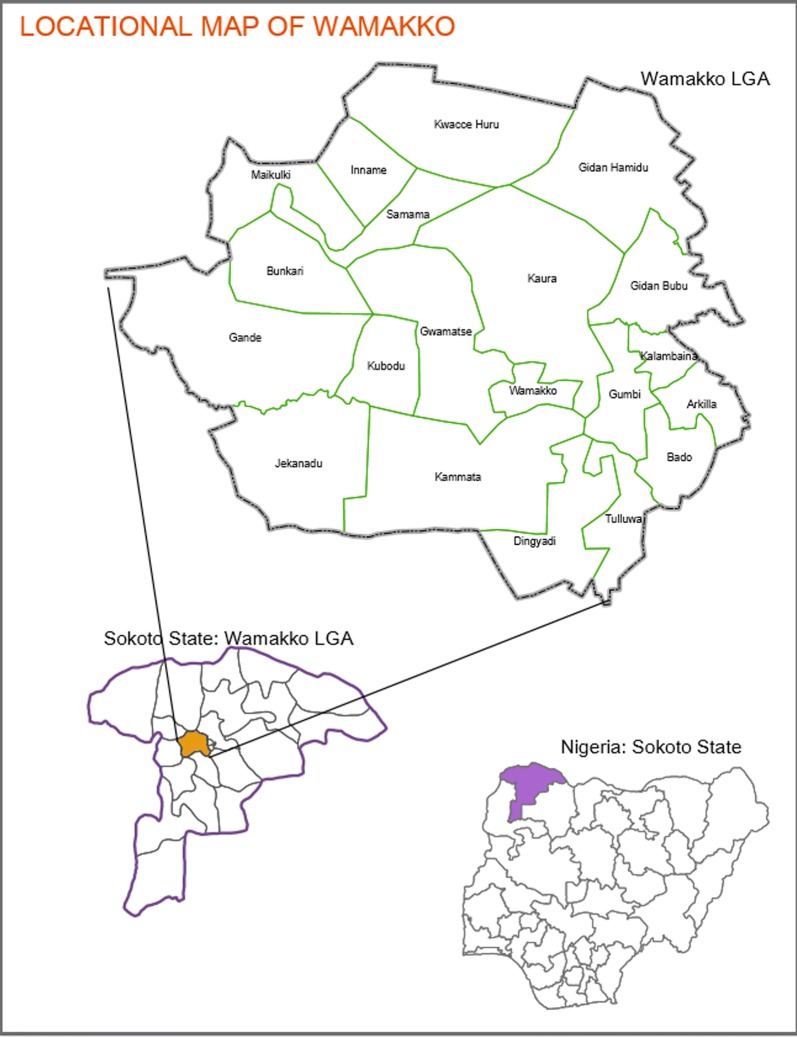
Locational map of Wamakko LGA in the context of Sokoto state and Nigeria

**Fig. 4:**
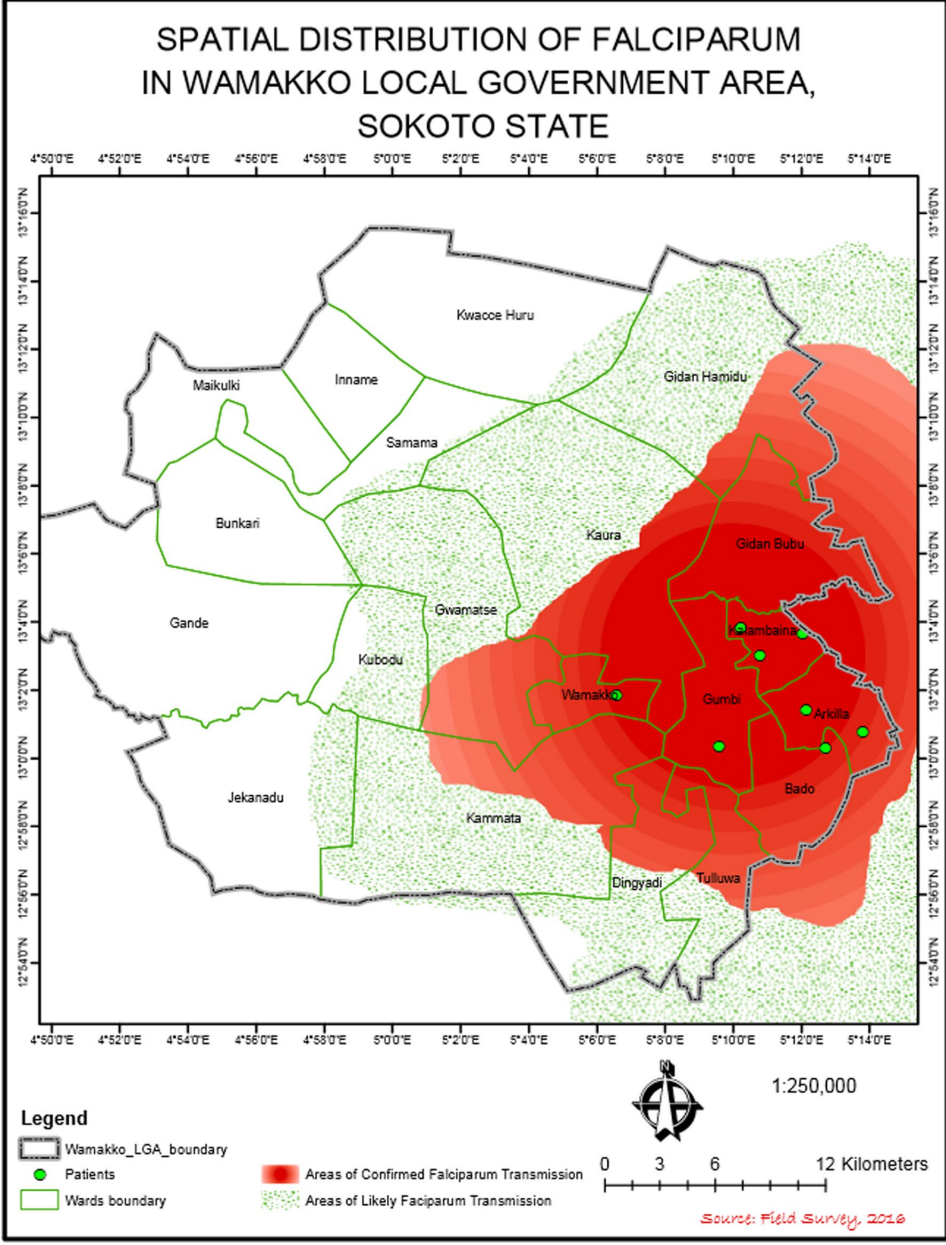
Map of Wamakko showing PfPR2–10

All the subjects with malaria parasitaemia irrespective of severity were found to have *P. falciparum* infection. The map generated shows an umbra region, which has a greater certainty and a penumbral region, which is less so. A locational map of Wamakko is shown in Fig. 3 and the map of transmission intensity is Fig. 4.

### Prevalence of malaria parasitaemia by season

**Fig. 5:**
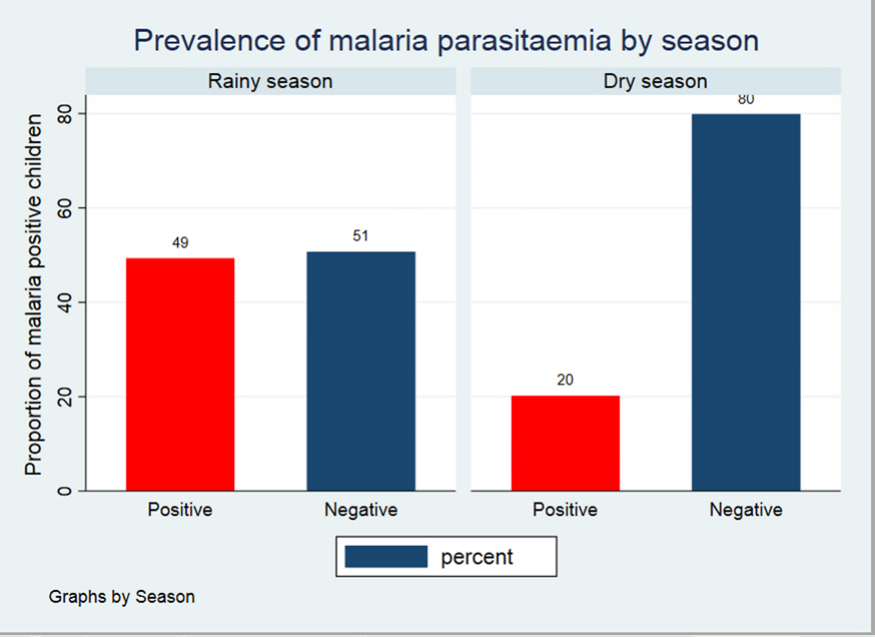
Prevalence of malaria parasitaemia by season

The prevalence of malaria was much higher in the rainy season than in the dry season as shown in Fig. 5.

### Age-specific parasite and spleen rates

**Fig. 6:**
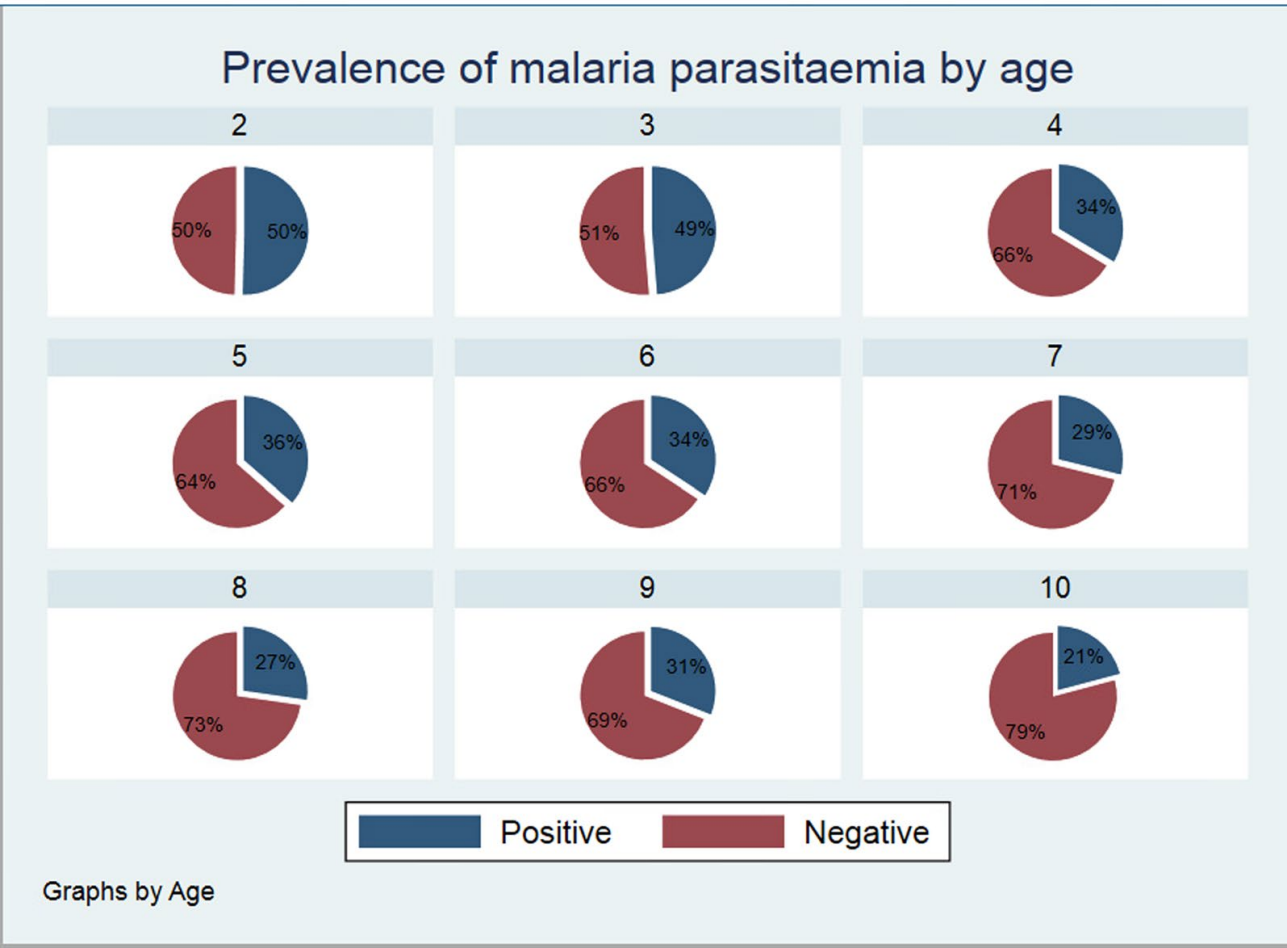
Prevalence of malaria parasitaemia by age

**Fig. 7:**
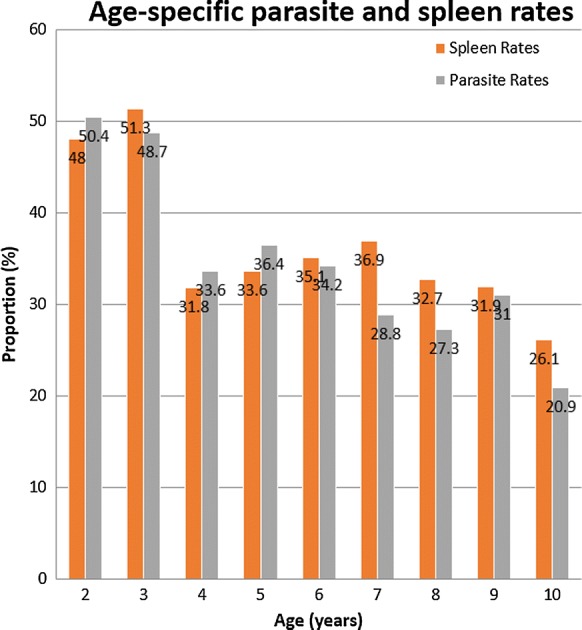
Age specific spleen and parasite rates

The prevalence of malaria was highest among children 3 years of age and lowest among those ages 10 years, as shown in Fig. 6. The age-specific spleen rates ranged from 26.1 to 51.3%. The highest spleen rate was seen among the children aged 3 years followed by those 2 years and lowest in those aged 10 years. There was a close relationship between the spleen rates and parasite rates as shown in Fig. 7.

The mean parasite count was higher in the rainy season than in the dry season. Although the counts were not normally distributed. The summary statistics are as shown in Table 3.

**Table 3:** Parasite count statistics for rainy and dry seasons

Season	Statistics				
	Arithmetic mean	Standard deviation	Geometric mean	95% confidence interval	
Rainy season	14,085	50,419	1921	1619	7804
Dry season	3460	22,008	894	695	1148

## Discussion

There are no community-based studies done within the study area to compare the findings of this study with, however, while the prevalence of malaria was found in this study to be 34.8%, the MAPs projections for Sokoto in 2015 which is modelled to show prevalence among the 2–10 year age group, was in the range of about 30% which is similar to the finding in this study [22]. The prevalence, when compared with serial malaria indicator studies performed in 2010 [16] and 2015 [23] shows a progressive reduction; from 48.1 to 37.1% for North Western Nigeria, and a prevalence of 46.6% for Sokoto in 2015. The prevalence in the MIS studies reflects the age range of 6 to 59 months which is probably higher than for the age included in this study because including the children from 6 to 10 years is likely to reduce the overall prevalence, as is the same likely consequence of excluding those aged 6 months to 2 years who generally have a higher prevalence rate [24]. This study suggests a trend of reducing endemicity as is common to most parts of Africa probably due to an intensification of malaria control efforts in Nigeria and the entire AFRO region of the WHO [25]. Serial National Demographic and Health Surveys (NDHS) and Malaria Indicator Surveys (MIS) have shown a progressive rise in the possession and use of insecticide treated nets and this may be one plausible reason for the decreasing endemicity. Ownership of at least one ITN rose steadily from 2% through 8% and 42% to 68.8% nationally in 2003 [26], 2008 [27], 2010 [16], 2013 [28] and 2015 [23], respectively. In the Northwest of Nigeria, it was 58.2%, 49.2% and 90.6% in 2010, 2013 and 2015, respectively [16, 23, 29]; and it rose from 56% in 2013 to 77.3% in 2015 in Sokoto state. In the same vein, use of ITNs by children under 5 years showed a steady rise nationally from 1 to 6%, 28.9 and 43.6 in 2003,2008, 2010 and 2015 respectively [16, 23, 27–29]. It was 61.9% in North-Western Nigeria in 2015, having risen from 28.9 in 2010 [16, 23].

The prevalence is also lower in comparison to 45.4% found in a study by Jiya et al. [30] in Sokoto between 2007 and 2009. Additionally, it was lower than the prevalence of 49.6% among children under the age of 5 years in the same study. Considering both age-specific prevalence rates, there is likely a definite reduction in the prevalence, even when the upper bound 95% CI of this study (37.8%) is considered. Despite the 2009 study being a hospital-based study, the prevalence for the current study is likely to be lower than the former. It is however slightly higher than the projected national average of 29% for 2015, with wide inter-regional differences [22]. The Nigerian Malaria Indicator Survey (MIS) of 2015 found a higher prevalence of 46.6% than this study, although the age of included subjects ranged from 6 to 59 months which will limit the comparability of results from this study due to the different age ranges of subjects [23]. The lower age group represented in the MIS is likely to give a higher prevalence than this study, although the margin of difference suggests that it is likely to be higher, despite this difference. The inclusion of subjects 6 months to 2 years who have been excluded in the current study is also likely to result in a higher prevalence because this group has an increased risk of malaria infection. This is because they are yet to gain significant immunity against malaria, as stipulated under the steady state assumption which postulates a higher rate of infection until 2 years of age when some immunity will be acquired and the incidence will then decline from 5 years onwards as the individuals become more immune [24]. Furthermore, the exclusion of the older age groups of 6–10 years from the national MIS is likely to have a similar effect because generally, these children are more immune and therefore at less risk of malaria. The results of the current study showed a similar albeit lower prevalence than in Abia and Plateau states in 2010 which showed parasite rates of 36.1% and 36.6%, respectively [31], although the inclusion of individuals older than 10 years and younger than 2 years limits the comparability to the present study. Both were also carried out in the month of September which is usually a month when malaria incidence has begun to decline. The prevalence in this study is also low when compared to Awka in the humid tropical rainforest belt of south-east Nigeria, where the prevalence was 58.2% in 2011 among children aged 1 to 10 years which is close to the age included in this study [32]. However, this is unsurprising, considering the vegetation of this region as compared to Sokoto where the present study took place [32]. The prevalence is also lower in comparison to the prevalence in Holmare settlement, in North Eastern Nigeria where the prevalence was found to be 38.4% among children aged 3 to 10 years in the entire hamlet. Although this value is similar to the upper bound 95% CI level from the present study, considering that confidence intervals were not reported by the authors of the Holmare study. The Holmare prevalence was, however, a single measure, late during the rainy season [33]. Exclusion of children 2 years of age is likely to have reduced the prevalence in this study, considering the highest age-specific prevalence was in this age group during the present study.

The only species of *Plasmodium* found in this study was *P. falciparum*. However, there was a difference in the prevalence determined using microscopy and RDT. This difference was not significant considering the overlap between the 95% confidence intervals-31.9% and 37.8% for microscopy and 30.9% and 36.8% for RDT. The positives by microscopy undetected by the RDT may be considered false negatives for the RDTs, which increase with lower levels of parasitaemia, particularly in the dry season. There could also be false positives which are unidentified by microscopy which are actually positive due to human error. Overall however, the kappa statistic indicates that both tests are comparable as used in this study.

Sokoto state was shown to have only minimal variation in climatological data between locations across the state, the only exceptions being along the basin of the Rima River, which has only Goronyo Local Government area of the state along its floodplain. The temperature differences between locations across the state at any point in time is 1.2 to 2 °C annually and rainfall only differs by about 12 mm even along the flood plains [14, 15]. The altitude is fairly stable across the state with uniform Sudan Savanna vegetation except along the Rima River Basin. These factors allow a generalizability of the average prevalence from Wamakko to the entire state with a variation expected only in November, towards the end of the rainy season [15]. This is likely because higher parasite rates are likely to linger around the river basin along the flood plains of the Sokoto-Rima River than in the other parts of the state. Considering a uniform projection across the state, a map was produced with the representation of prevalence areas for Wamakko LGA. The map has an advantage over the previous maps because it provides true estimates rather than projections of the malaria transmission intensity. The applicability of the map could potentially extend beyond malaria to other vector-transmitted diseases, for which the vector behaviour is known. A comparison of the prevalence of schistosomiasis between the areas extending to 5 km around water bodies and other parts in Kebbi state by Jega RM (unpublished) suggests that the prevalence and ‘hot spots’ for schistosome infection can been mapped and play a critical role in the planning of interventions. This is converse for the other maps which are specifically tailored to malaria transmission. The main weakness of this map is that where there is no data available, it will be impossible to map the transmission intensity. However, this lack of data is unlikely to affect malaria mapping, evidenced by the numerous data points used for the MAP. It will also ensure that any map produced is accurate, unlike the MAP, which compromises its accuracy when the data points are inadequate. Having a penumbra and umbra region in the new map also helps decision-making, in which case an intervention can be planned with more emphasis on the umbra region.

The parasite rates were much higher at the younger ages of 2 years and 3 years as expected; due to a lower degree of acquired malarial immunity at these ages. It was however not very patterned thereafter. This finding is in keeping with previous MIS reports and findings by Abdullahi et al. [34] in Sokoto in 2009. However, in Abia and Plateau states of Nigeria in 2010, it was higher in the age group of 5 to 9 years of age, although the prevalence was correlated with age. This 5 to 9 years age group could show a higher prevalence in a setting of higher ITN use, as younger children were more likely to sleep under ITNs with their mothers as demonstrated in MIS studies in Nigeria [16]. One study however showed a higher rate of ITN use and higher preventive efficacy in the 5 to 9 year age group [35].

Higher spleen rates were found in children less than 5 years of age, suggesting a more robust immunologic response against malaria by under-fives. The age-specific spleen rate mirrored that of the parasite rates and was highest for children aged 2 and 3 years with a statistically significant difference among the ages. The spleen rates were consistently higher than the parasite rates across all ages.

One striking observation for the parasite counts was that although the mean parasite rate was higher for the rainy than the dry season, the data was not normally distributed, expectedly because the range is very broad and the zero counts were predominant.

## Limitation

The study was a two-point study due to constrained resources and as such did not completely show the trend over a year, although this is well established from previous studies.

## Conclusions

- The prevalence and endemicity of Wamakko are similar to MAPs predictions and may be generalizable to Sokoto state based on Malaria Atlas Project projections.
- For planning purposes, the projections may be utilized, with more efforts at validation of the MAPs in other locations and terrains.
- It is feasible to obtain a season-sensitive map for malaria endemicity and prevalence to be applied locally within Nigeria and beyond.

## Data Availability

The datasets generated and/or analysed during the current study are available in the figshare repository, 10.6084/m9.figshare.11590542.v1.

## Abbreviations

AFRO: African region of WHO
API: Annual parasite incidence
CI: Confidence interval
CRF: Case report form
EIR: Entomologic inoculation rate
GIS: Geographic information systems
GPS: Global Positioning System
ITN: Insecticide-Treated Net
LGA: Local government area
MAP: Malaria Atlas Project
MARA: Mapping Malaria Risk in Africa
MIS: Malaria Indicator Survey
NDHS: National Demographic and Health Survey
PfPR2–10: *Plasmodium falciparum* prevalence rate among 2 to 10 year olds
RDT: Rapid diagnostic test
SSA: sub-Saharan Africa
WHO: World Health Organization
